# Digital marketing by big bubble tea brands in China in 2023: a content analysis

**DOI:** 10.1017/S1368980025101110

**Published:** 2025-10-06

**Authors:** Lin Wang, Navoda Nirmani Liyanapathirana, Florentine Martino, Jia Ying Wu, Mengshan Ren, Kathryn Backholer

**Affiliations:** 1 Global Centre for Preventive Health and Nutrition, Deakin University, Geelong, VIC, Australia; 2 State Key Laboratory of Media Convergence and Communication, Communication University of China, Beijing, China; 3 Graduate School, Communication University of China, Beijing, China

**Keywords:** Bubble tea brand, Digital marketing, Bilibili, Social media

## Abstract

**Objective:**

Bubble tea is known to have adverse health impacts due to its high sugar content. However, the influence of digital marketing on its consumption, especially among young people, remains unclear. This study aimed to describe the digital marketing strategies of Chinese bubble tea brands.

**Design:**

A content analysis of all marketing posts made by the top three Chinese bubble tea brands (by market share) – XIXUE, HEYTEA and NAYUKI – on Bilibili between 1 January 2023 and 31 December 2023.

**Setting:**

Bilibili, a popular social media platform among Chinese young people, in 2023.

**Participants:**

Not applicable.

**Results:**

Branding is central to the digital marketing strategies of bubble team brands, with the majority of posts using brand logos (99 %), branded effects (80·1 %) and branded characters (63 %), including children’s characters (19 %). Marketing strategies promoting user interaction were also common, reflected in the frequent use of hashtag campaigns (63 %), general engagement strategies (43 %) and competitions (10 %). Cultural elements that are integrated into the marketing message to resonate with the audience’s cultural identity were present in 47 % of posts.

**Conclusions:**

Bubble tea brands are using a range of digital marketing strategies to engage consumers and build brand presence in the competitive bubble tea market in China. Measures to protect young consumers from the exposure of such marketing should be considered as a way of improving population diets and reducing excess weight gain.

Unhealthy diets are a leading cause of global mortality and morbidity^(1)^. Regular consumption of sugary drinks has been found to be the single leading cause of excess weight gain, particularly among children, and is directly associated with increased risks of numerous chronic conditions, including type 2 diabetes, CVD, dental caries and some cancers^(2–4)^. Artificial sweeteners are often used as a sugar substitute, but may also pose health risks, such as excess weight gain, overweight and obesity^(5)^. Although the consumption of sugar and artificial sweeteners is reported to be relatively low in China^(6)^, they have nonetheless been identified as a major contributor to the rapid increase of overweight and obesity among Chinese children^(7)^.

In recent years, bubble tea – a tea-based beverage featuring chewy tapioca balls and various toppings such as grass jelly, aloe vera, red bean and popping boba^(8,9)^ has gained worldwide popularity for its unique texture and customizable flavours^(10)^. According to iiMedia Research, the Chinese bubble tea market was valued at 279·59 billion yuan in 2021 and is projected to reach 374·93 billion yuan by 2025^(11)^. A market research survey conducted in April 2023 found that 61 percent of respondents in China aged 16–24 years reported that they typically order Bubble Tea through food delivery apps^(12)^. Bubble tea often contains high levels of sweeteners, artificial additives and other ingredients, with studies linking high consumption to overweight among school-age children^(13)^. Additionally, high frequency of bubble tea consumption has been significantly associated with an increased risk of depression and anxiety symptoms among Chinese young adults, likely due to the high sugar, caffeine and trans-fatty acid content in bubble tea, which negatively impacts mental health^(8)^.

International evidence shows that children and young people are exposed to, and engage with, high levels of marketing of unhealthy products that are high in fat, sugar and salt across multiple digital channels^(14)^. Empirical evidence indicates that marketing of unhealthy foods and beverages can significantly influence consumer preferences, purchasing patterns and consumption levels^(15)^. Accordingly, the WHO strongly advocate for governments to prohibit the marketing of such products^(16)^. In China, whilst the Advertising Law of the People’s Republic of China sets general standards for advertising practices, these are primarily focused on prohibiting false or misleading content and do not distinguish between healthy or less healthy foods or beverages. Similarly, the Food Safety Law of the People’s Republic of China is limited to restricting the marketing of therapeutic claims or use of medical terminology on foods and beverages.

Leading bubble tea brands in China, such as XIXUE, HEYTEA and NAYUKI, have embraced advanced digital marketing strategies, utilising social media, e-commerce and mobile apps for brand promotion and consumer engagement. Despite the extensive use of these techniques, there is a lack of systematic examination of their digital marketing practices. Understanding these practices is crucial for advocates and policymakers to develop policies that counteract the harmful effects of pervasive unhealthy food and beverage marketing.

Bilibili, a popular social networking platform among Chinese youth, allows users to create, post, watch and engage with videos. Data from Bilibili indicate that those born between 1990 and 2009 (15–35 years old today) account for nearly 80 % of its user base in 2023. This youthful and vibrant community on Bilibili is a prime target for brand promotion strategies, especially for traditional brands aiming to connect with younger demographics.

We aimed to quantify and examine the nature of bubble tea marketing on China’s most popular social media platform, Bilibili. By doing so, we sought to describe the marketing practices that have contributed to the popularity of the three top bubble tea brands in China.

## Methods

We identified and conducted a content analysis of all marketing posts made by the top three bubble tea brands (by market share) in China on Bilibili between 1st of January 2023 and 31st of December 2023. The detailed methodology is described in subsequent sections.

### Description on social media platform

Bilibili is an online community and video sharing platform in China. Bilibili videos can span diverse topics such as animations, games, music or lifestyle. Each video is paired with an attention-grabbing cover (thumbnail) and a concise, compelling title. Creators often include detailed descriptions beneath the video, along with hashtags to improve discoverability. User interaction features include real-time bullet comments and comment sections, and likes, coins favourites and sharing options help support creators and amplify content visibility. The user demographic is predominantly young people, with a high degree of interactivity between content creators and viewers. Watching and browsing videos on Bilibili does not require a login account, but posting comments and engaging in various forms of interaction do require an account. The three bubble tea brands selected in this study each have their own independent accounts and pages.

### Data collection

The top three bubble tea brands in China were selected based on popularity and market share. These included HEYTEA (with 44·14 % of bubble tea consumers reporting they frequently purchase this brand), NAYUKI (41·45 % consumers) and MIXUE (35·45 % consumers)^(17)^.

For each bubble tea account on Bilibili, we recorded the numbers of followers and videos posted by 31 December 2023 and the date of the first ever post on the Bilibili platform. We also extracted all posts (all posts are videos) made by each of the brands between 1st January and 31st December 2023 and recorded the date of post and the numbers of views, likes and comments. We coded video content against a pre-specified coding guide adapted from a study examining unhealthy food and non-alcoholic beverages marketing on Tiktok (another video short video social media platform) using a dichotomous (‘yes/no’) classification for each coding element (Table 1)^(18)^. The guide from the prior study was selected for its inclusion of a comprehensive range of marketing strategies, however, we added ‘cultural elements’ to the coding guide to capture use of traditional cultural elements in digital marketing by Chinese brands, reflecting emerging digital marketing trends^(19)^.

**Table 1.** Coding guides for analysis of owned media content on Bilibili

Code	Description
Branding	Whether branding, such as logos, colours, fonts, trademarks or slogans is present, including branding in product images and branded effects.
Product image	Whether product images are present, including packaged product images and unpackaged product images where the product is clearly identifiable as the brand’s product.
Branded effects	Whether a branded effect (e.g. stickers, filters or special effects that feature the brand and appear within Bilibili for users to add to videos) is present.
Corporate social responsibility or philanthropy	Whether a statement of an ethical or sustainability standpoint or initiative or partnership with a charity is present.
Celebrities/influencers	Whether anyone with a media profile or a verified badge (indicating that Bilibili has confirmed the account belongs to the user it represents), excluding professional sportspeople, is present.
Sportspeople	Whether a professional sportsperson is present.
Children’s characters	Whether a third-party cartoon or character is present.
Branded characters	Whether a cartoon or character developed by the brand is present.
Price promotions	Whether a price promotion (e.g. promotion of regular prices or promotions of limited time offers, discount menus, 2 for 1 deals) is present.
Competitions	Whether a competition is promoted.
Engagement	Whether interaction or conversation is promoted.
Hashtag campaigns	Whether a brand engages in a hashtag challenge as a means of promotion. A hashtag challenge is where Bilibili encourages users to create video content performing a certain task and to tag their video or image using a given hashtag. Content created as part of the hashtag challenge is then algorithmically boosted by Bilibili, increasing exposure to a given post.
Sponsorships or partnerships	Whether a statement of sponsorship or partnership with an event, other brand or organization is present, excluding any already captured by ‘corporate social responsibility or philanthropy’
Cultural elements	Whether the presence of Chinese traditional cultural elements within the video content, including but not limited to, traditional festivals, cuisine, popular city landmarks and cultural symbols (e.g. such as tea, cultural figures, classical poetry and iconic films).

To ensure reliability of data coding, two authors (WL and WJY) independently coded all posts using the standardised coding scheme as described above. After completing the independent coding, the two authors engaged in cross-coding, which involved checking each other’s coding to identify and resolve any potential discrepancies. This process included discussing points of disagreement until consensus was reached, thereby enhancing the consistency of the coding.

During the cross-coding process, a total of eleven discrepancies were identified. Specifically, MIXUE had ten instances of disagreement, HYTEA had none and NAYUKI had one. The discrepancies were primarily related to two areas: three instances concerned ‘branded characters or children’s characters,’ and eight instances were related to ‘corporate social responsibility or philanthropy’ and ‘sponsorships or partnerships.’ To resolve these discrepancies, we engaged in discussions and collectively re-watched the videos, strictly applying the definitions from Table 1 to determine the type of each tag, which ultimately led to a consensus.

### Data analysis

We calculated descriptive statistics using Microsoft Excel. For every brand, the median and range of likes, comments and shares per video and the percentage of videos using each marketing strategy were calculated by category and in total.

## Results

All three bubble tea brands created accounts on Bilibili and posted their first videos between 2019 and 2020. As of 31 December 2023, MIXUE had the largest following with over 1·3 million followers and had shared 382 videos, amassing almost 13 million cumulative likes (see Table 2). NAYUKI had the fewest followers (*n* 217 000) and the lowest engagement, with 657 000 cumulative likes of posted videos. For every brand, the median and range of likes, comments and shares per video and the percentage of videos using each marketing strategy were calculated in total and by marketing strategies listed in Table 1.

**Table 2.** Details of three bubble tea brand accounts on Bilibili (as of 31 December 2023)

Brand	Followers, *n*	Videos posted, *n*	Date first video posted	Cumulative likes of videos, *n*
MIXUE	1 333 000	382	30-3-2020	12 905 000
HEYTEA	280 000	161	27-8-2019	1 737 000
NAYUKI	217 000	276	1-6-2020	657 000

**Figure 1.**
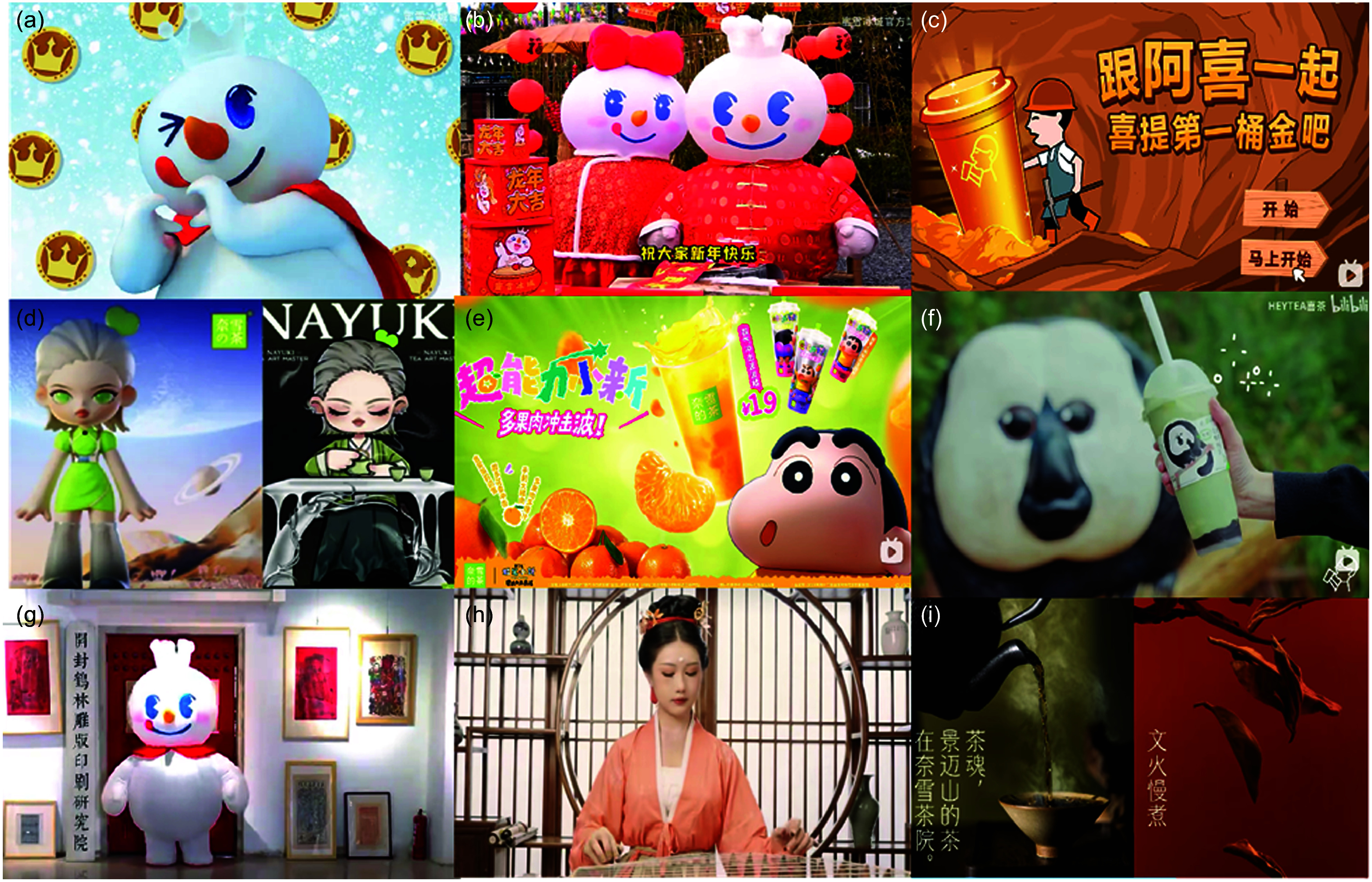
Examples of marketing strategies used by the three leading bubble tea brands on the Chinese social media platform, Bilibili. (a) MIXUE using animated special effects with its branded characters, (b) MIXUE branded characters, (c) HEYTEA branded characters, (d) NAYUKI branded characters, (e) NAYUKI using children’s characters, (f) HEYTEA using product images, (g) MIXUE using cultural elements of Chinese Intangible Cultural Heritage: Woodblock Painting, (h) HEYTEA using cultural elements of traditional clothing, (i) NAYUKI using cultural elements of tea.

In 2023, MIXUE posted the greatest number of videos on BiliBili, releasing 159 in the twelve-month period, with a median of 76 000 views, > 5500 likes and 205 comments per post. HEYTEA posted least frequently (38 posts in 2023), but engagement was relatively high, with a median of 34 000 views per video, 1588 likes and 62 comments per post. Among the three brands, the most frequently used marketing strategies were branding, branded effects and the use of branded characters. Branding appeared in nearly every post, a small percentage of MIXUE posts (1·3 %) contains more generic content relating to bubble tea without featuring any forms of brands but a majority of posts did include some form of branding, as stated in above. While branded effects were used in 80·1 % of posts across all bubble tea brands, with HEYTEA utilising them the most frequently (94·7 %) and MIXUE the least (74·2 %). For example, MIXUE used branded effects by creating a theme song using animated special effects with its branded characters (see examples in Figure 1(a)). Branded characters featured in 63·2 % of all posts, with MIXUE using branded characters most frequently, using them in 87·4 % of all posts (compared with 21·2 % for HEYTEA and 28·1 % for NAYUKI; see examples of how branded characters were used in Figure 1(b)–(d). Use of children’s characters was used most frequently by NAYUKI (34·4 %) (see examples in Figure1(e)). Product imagery was used in 59·8 % of posts across the three brands, with HEYTEA (94·7 %) and NAYUKI (87·5 %) using them most frequently (see examples in Figure 1(f)).

Other frequently used strategies included engagement techniques, cultural elements, sponsorships or partnerships, children’s characters and celebrities/influencers. Across the three brands, 42·9 % of all posts included an engagement strategy, with NAYUKI using this marketing strategy most frequently (84·4 %) compared with HEYTEA (47·4 %) and MIXUE (25·2 %). For instance, NAYUKI engaged its audience by conducting lotteries within the comment section, selecting lucky viewers to receive discount coupons. Additionally, the brand incorporates voting links midway through videos to encourage viewer interaction. In NAYUKI’s series of videos showcasing regional cuisines, fans are invited to share their hometown specialties in the comment section, fostering a sense of community and further enhancing engagement. Both HEYTEA and NAYUKI frequently used cultural elements (see examples in Figure 1(g)–(i)), with usage rates of 50 % and 67·2 %, respectively. MIXUE on the other hand used sponsorships or partnerships strategies in a large proportion of their posts (44 %).

The least frequently used strategies were competitions (9·7 %), corporate social responsibility (CSR) or philanthropy (2·7 %), special price promotions (2·3 %) and sportspeople (0 %).

Hashtag campaigns were a popular strategy that the bubble tea brands engaged with as a means of promotion. Hashtag campaigns are typically initiated by the Bilibili platform, which encourages users to create content using a specific hashtag, often with a theme or challenge. Content created using the hashtag can then be boosted to expand the reach of a post. Across the 12-month period MIXUE, HEYTEA and NAYUKI used a Bilibili hashtag in 53·5 %, 57·9 % and 68·8 % of posts, respectively (see Table 3). For example, the ‘Humor Research Institute’ (搞笑研究所) hashtag challenge resulted in thirty-eight videos from MIXUE, five from HEYTEA and eight from NAYUKI utilising this hashtag. The hashtag challenge encouraged users to create content to showcase their sense of humour and creativity. In one post in response to this hashtag challenge, MIXUE had its branded character dance in a video, which garnered 40 000 views. Use of the Bilibili hashtag appeared to amplify the reach of the post MIXUE but not for HEYTEA or NAYUKI. For MIXUE posts using a Bilibili hashtag were viewed with a median of 121 000 times (with a median of 7241 likes and 221 comments), compared with all posts by MIXUE, which were viewed a median of 76 000 times (with a median of 5508 likes and 205 comments. For HEYTEA and NAYUKI, the median views and likes were less than the total sample of posts (see Tables 3 and 4).

**Table 3.** Analysis of top three bubble tea brand’s posts on Bilibili in the 12 months between 1st of January and 31st of December 2023

	MIXUE		HEYTEA		NAYUKI		Overall	
Reach								
Number of post in 2023	159		38		64		261	
Median views per post	76 000		34 000		31 500		34 000	
Median likes per post	5508		1588		835		1588	
Median comments per post	205		62		297		62	
	*n*	%	*n*	%	*n*	%	*n*	%
Marketing strategies (number (%))								
Branding	157	98·7 %	38	100·0 %	64	100·0 %	259	99·0 %
Product images	64	40·3 %	36	94·7 %	56	87·5 %	156	59·8 %
Branded effects	118	74·2 %	36	94·7 %	55	85·9 %	209	80·1 %
Corporate social responsibility or philanthropy	5	3·1 %	0	0 %	2	3·1 %	7	2·7 %
Celebrities/influencers	34	21·4 %	5	13·2 %	3	4·7 %	42	16·1 %
Sportspeople	0	0 %	0	0 %	0	0 %	0	0 %
Children’s characters	17	10·7 %	11	29 %	22	34·4 %	50	19·2 %
Branded characters	139	87·4 %	8	21·1 %	18	28·1 %	165	63·2 %
Special price promotions	3	1·9 %	1	2·6 %	2	3·1 %	6	2·3 %
Competitions	5	3·1 %	1	2·6 %	20	31·3 %	26	9·7 %
Engagement	40	25·2 %	18	47·4 %	54	84·4 %	112	42·9 %
Hashtag campaigns	85	53·5 %	22	57·9 %	44	68·8 %	151	57·9 %
Sponsorships or partnerships	70	44 %	6	15·8 %	8	12·5 %	84	32·2 %
Cultural elements	40	25·2 %	19	50 %	43	67·2 %	102	39·1 %

**Table 4.** Description of the hashtag campaigns that the three bubble tea brands engaged with on Bilibili

Brand	Engagement with unique hashtag campaigns, *n*	Median views, *n*	Median likes, *n*	Median comments, *n*
MIXUE	22	121 000	7241	221
HEYTEA	13	23 000	51	753
NAYUKI	15	27 000	305	752

## Discussion

Our study presents the first comprehensive analysis of the digital marketing strategies utilised by China’s leading bubble tea brands – XIXUE, HEYTEA and NAYUKI – on Bilibili in 2023. Our findings indicate that these brands place a strong emphasis on creating a distinct brand image by integrating branding, brand effects and branded characters. They also strategically incorporate cultural elements into their marketing posts, such as traditional tea and Chinese culture, to appeal to local audiences and attempt to enhance the visibility of their marketing posts through the use of Bilibili hashtag campaigns.

Our finding that bubble tea brands consistently used branding in their marketing messages aligns with prior international research. For example, a 2021 Australian study analysing the marketing content of the top sixteen food and beverage brands on Tik Tok found that 87 % of videos posted by these brands included branded content^(19)^. Similarly, a 2017 Australian study examining the marketing posts by the most popular unhealthy food brands on Instagram found that use of branding elements was one of the most common marketing strategy used^(20)^. Bubble tea brands not only design branded characters but also incorporate them as logos, colours and slogans across their posts. Additionally, they frequently use branded effects in their videos, such as stickers, filters or special effects, to enhance the charming or humorous qualities of their branded characters. This strategy, which endows products with a captivating and child-friendly aesthetic, leverages their appeal to resonate with children and young people^(21)^. Empirical studies have consistently shown that the inclusion of brand mascots in the marketing of unhealthy snacks can significantly boost children’s engagement, bolster brand recognition and cultivate a favourable perception of the brand^(22–24)^. Our study indicates that this strategy is particularly prevalent in the Chinese bubble tea industry.

Our research also identified the frequent use of traditional cultural elements in bubble tea marketing, a finding unique to our study. Previous research has demonstrated the incorporating Chinese cultural elements into global brands positively influence consumer purchase intent, and this effect is moderated by consumer cultural identity, emphasizing the importance of cultural considerations in marketing strategies for China^(25)^. Bubble tea brands on Bilibili integrate cultural elements, such as traditional Chinese tea culture and intangible cultural heritage, into their marketing strategies, resonating with local audiences and potentially enhancing brand identity.

We also found that the bubble tea brands actively engage with Bilibili hashtag campaigns, leveraging the platform’s algorithmic promotion of hashtag content to boost the visibility of their posts. However, only one bubble tea brand appeared to experience an increase in views and likes from posts utilizing a hashtag from a Bilibili hashtag challenge. Nevertheless, engaging with hashtag challenges may still be a strategic marketing technique by bubble tea brands to connect with users by demonstrating that they are in touch with popular trends. A prior study investigating the impact of TikTok’s hashtag challenge found that brand sentiment in hashtag challenge was generally positive, with 50·8 % of respondents expressing a favourable view and 32·5 % of users reporting that they had purchased products advertised through the hashtag challenge^(26)^.

Our findings indicate that bubble tea brands rarely use CSR initiatives or sports celebrity endorsements in their marketing strategies. This contrasts with similar studies conducted in Western countries, where food and beverage companies often integrate CSR into their marketing strategies to enhance brand image, build consumer trust and differentiate products^(27)^. For example, in Australia, major food companies have been found to use CSR initiatives related to the environment and consumer responsibility to build brand image, targeting parents and children through community activities and aligning with respected organisations and events to transfer positive image attributes to their brands^(28)^. A 2023 Slovakian study found that consumers view CSR as a marketing tool that improves company reputation and image^(29)^. International research also shows that food and beverage companies use sports celebrity endorsements to effectively enhance brand recognition and influence consumer behaviour, particularly among youth^(30)^. The absence of these strategies by bubble tea companies in our study may reflect a focus on taste and experience, with their target market – mainly children and teenagers – who may be more attracted to branded characters rather than CSR and celebrity endorsements.

The findings from our study on the digital marketing strategies of leading bubble tea brands in China have implications for public health policy. The extensive use of branded content, including branded characters, and engagement strategies appeal with younger audiences, potentially influencing their consumption in ways that contribute to unhealthy dietary patterns^(31)^. With bubble teas containing high levels of sugar and artificial sweeteners and the international evidence showing that unhealthy food marketing negatively influences dietary intakes^(16)^, these marketing strategies may be contributing to the rapid increase of overweight and obesity among Chinese children^(7)^. This underscores the need for regulatory measures to protect children and young people in China from the digital marketing of unhealthy foods and beverages. Chile has implemented regulations that restrict all marketing on unhealthy foods and beverages that is directed to children,^(32)^ and the UK Government has adopted a law to ban all ‘paid’ marketing of unhealthy foods and beverages online^(33)^. Implementing similar regulations in China could potentially curb the influence of such marketing strategies, thereby contributing to better public health outcomes.

While this study provides a comprehensive analysis of the digital marketing strategies used by China’s leading bubble tea brands on Bilibili, several limitations should be acknowledged. First, the scope of the study is limited to a single social media platform, Bilibili. Future research could expand the analysis to include other popular social media platforms such as WeChat, Douyin (TikTok) and Xiaohongshu (Little Red Book) to provide a more holistic view of the digital marketing strategies used by bubble tea brands. Additionally, this study focuses on only three major bubble tea brands – XIXUE, HEYTEA and NAYUKI. Including a broader range of bubble tea brands in future research could offer more comprehensive insights into the industry’s marketing practices and trends. Lastly, we did not explore the impact of individual users’ reviews and personal experiences shared on social media. Future research could investigate how user-generated content influences consumer perceptions and brand loyalty in the bubble tea industry.

### Conclusion

Our study provides the first comprehensive analysis of how leading Chinese bubble tea brands leverage digital marketing on the popular social media platform, Bilibili. The heavy use of branding, cultural elements and platform-specific engagement strategies reflects international trends in food and beverage marketing, while also highlighting unique local approaches. Given the high sugar content of bubble tea and the established impact of digital food marketing on dietary choices^(34)^, these strategies may contribute to unhealthy consumption patterns, particularly among children and young people in China. Government-led regulations restricting unhealthy food and beverage marketing on digital platforms can protect young people from unhealthy diets and excess weight gain. Future research should examine marketing strategies across multiple platforms and brands, as well as the role of user-generated content in shaping consumer perceptions.
